# Differential roles of uterine epithelial and stromal STAT3 coordinate uterine receptivity and embryo attachment

**DOI:** 10.1038/s41598-020-72640-0

**Published:** 2020-09-23

**Authors:** Takehiro Hiraoka, Yasushi Hirota, Yamato Fukui, Mona Gebril, Tetsuaki Kaku, Shizu Aikawa, Tomoyuki Hirata, Shun Akaeda, Mitsunori Matsuo, Hirofumi Haraguchi, Mayuko Saito-Kanatani, Ryoko Shimizu-Hirota, Norihiko Takeda, Osamu Yoshino, Tomoyuki Fujii, Yutaka Osuga

**Affiliations:** 1 Department of Obstetrics and Gynecology, Graduate School of Medicine, The University of Tokyo, 7-3-1 Hongo, Bunkyo-ku, Tokyo, 113-8655 Japan; 2 Department of Obstetrics and Gynecology, Kitasato University, Sagamihara, Kanagawa Japan; 3 Frontier Outstanding Research for Clinical Empowerment (FORCE), Japan Agency for Medical Research and Development (AMED), Bunkyo-ku, Tokyo, 113-8655 Japan; 4 Department of Internal Medicine, Center of Preventive Medicine, School of Medicine, Keio University, Shinjuku-ku, Tokyo, Japan; 5 Center for Molecular Medicine, Jichi Medical University, Shimotuke, Tochigi Japan

**Keywords:** Disease model, Reproductive disorders, Experimental models of disease, Endocrine reproductive disorders, Infertility

## Abstract

Although it has been reported that uterine signal transducer and activator of transcription 3 (STAT3) is essential for embryo implantation, the exact roles of uterine epithelial and stromal STAT3 on embryo implantation have not been elucidated. To address this issue, we generated *Stat3*-floxed/*Ltf-iCre* (*Stat3*-eKO), *Stat3-*floxed/*Amhr2-Cre* (*Stat3*-sKO), and *Stat3*-floxed/*Pgr-Cre* (*Stat3*-uKO) mice to delete *Stat3* in uterine epithelium, uterine stroma, and whole uterine layers, respectively. We found that both epithelial and stromal STAT3 have critical roles in embryo attachment because all the *Stat3*-eKO and *Stat3*-sKO female mice were infertile due to implantation failure without any embryo attachment sites. *Stat3*-eKO uteri showed indented structure of uterine lumen, indicating the role of epithelial STAT3 in slit-like lumen formation in the peri-implantation uterus. *Stat3*-sKO uteri exhibited hyper-estrogenic responses and persistent cell proliferation of the epithelium in the peri-implantation uterus, suggesting the role of stromal STAT3 in uterine receptivity. In addition, *Stat3*-uKO female mice possessed not only the characteristic of persistent epithelial proliferation but also that of indented structure of uterine lumen. These findings indicate that epithelial STAT3 controls the formation of slit-like structure in uterine lumen and stromal STAT3 suppresses epithelial estrogenic responses and cell proliferation. Thus, epithelial and stromal STAT3 cooperatively controls uterine receptivity and embryo attachment through their different pathways.

## Introduction

Implantation failure is a major problem of patients with infertility undergoing in vitro fertilization and embryo transfer (IVF-ET). Successful embryo implantation necessitates an intimate crosstalk between the receptive uterus and the implantation-competent blastocyst. These molecular and cellular interactions initiate when embryonic development to blastocyst stage is synchronized with the endometrium being receptive^1–3^. Although the molecular mechanism of uterine receptivity is not fully clarified, several molecules and pathways have been identified as key regulators of embryo implantation such as progesterone (P_4_) signaling, leukemia inhibitory factor (LIF), signal transducer and activator of transcription 3 (STAT3) signaling^3–7^.

STAT3 is a major transcription factor which transduces signals from interleukin (IL)-6 family cytokines including IL-6, IL-11, LIF, oncostatin M (OSM) and other growth factors^8^. LIF is crucial for embryo implantation and activates uterine STAT3 pathway^5,9^. Depending on the ligands and cell types, STAT3 exerts a variety of functions: for example, cell proliferation/differentiation and amplification/suppression of inflammatory responses^8,10^. In the process of acute and chronic inflammation, pro-inflammatory IL-6 produced by immune cells and fibroblasts activates STAT3 and induces a series of genes responsible for cell proliferation and inflammation^11^. At the same time, STAT3 also mediates the signal from anti-inflammatory IL-10 to suppress inflammation^12^. Since systemic *Stat3* null mice show embryonic lethality^13^, a conditional knockout mouse model of STAT3 was recently established to investigate the function of STAT3 in the uterus^14,15^. *Pgr-Cre* mice show the specific expression of Cre recombinase in the whole uterine layers^16^, and *Pgr*-Cre mice were crossed with *Stat3*-floxed mice^17^ to create *Stat3*-deleted mice at the whole uterine layers (*Stat3*-floxed/*Pgr-Cre* (*Stat3*-uKO)). *Stat3*-uKO female mice showed infertility due to implantation failure with enhanced estrogenic responses, presenting increased expression of 17β-estradiol (E_2_) -responsive genes *Ltf* and *Muc1* and persistent epithelial proliferation in the peri-implantation uteri. These findings indicate the role of uterine STAT3 in uterine receptivity by modulating E_2_ signaling, a critical determinant of uterine receptivity. In addition, our group recently demonstrated the key role of uterine STAT3 in uterine regeneration using *Stat3*-uKO mice^15^, suggesting that STAT3 acts on the physiological uterine reconstruction processes such as menstruation and postpartum. Thus, *Stat3*-uKO mice have been useful for the investigation of the role of uterine STAT3.

Uterine epithelium and stroma are two major components of the endometrium, and their interactions are involved in the process of the receptivity acquisition^1,18,19^. Uterine epithelium and stroma have independent and mutual functions to support uterine receptivity and embryo implantation^1,18–20^. Although the previous literature demonstrated the significance of uterine STAT3 in embryo implantation^14^, the role of epithelial and stromal STAT3 in the process of embryo implantation remains unclear. In this study, we generated *Stat3*-floxed/*Ltf-iCre* (*Stat3*-eKO) and *Stat3*-floxed/*Amhr2-Cre* (*Stat3*-sKO) mice to delete *Stat3* in the uterine epithelium and stroma, respectively, and investigated the detailed functions of epithelial and stromal STAT3 during embryo implantation. *Stat3*-eKO and *Stat3*-sKO female mice were infertile due to implantation failure without any embryo attachment sites. *Stat3*-eKO mice compromised the formation of slit-like structure in uterine lumen which enables normal embryo attachment. In contrast, *Stat3*-sKO mice showed epithelial hyper-estrogenic responses and persistent proliferation which impair uterine receptivity. In addition, *Stat3*-uKO mice showed both compromised luminal structure and persistent epithelial proliferation. Taken together, epithelial and stromal STAT3 supports uterine receptivity and embryo attachment through the regulation of different pathways.

## Results

### STAT3 is activated both in the epithelium and stroma of the peri-implantation uterus

**Figure 1:**
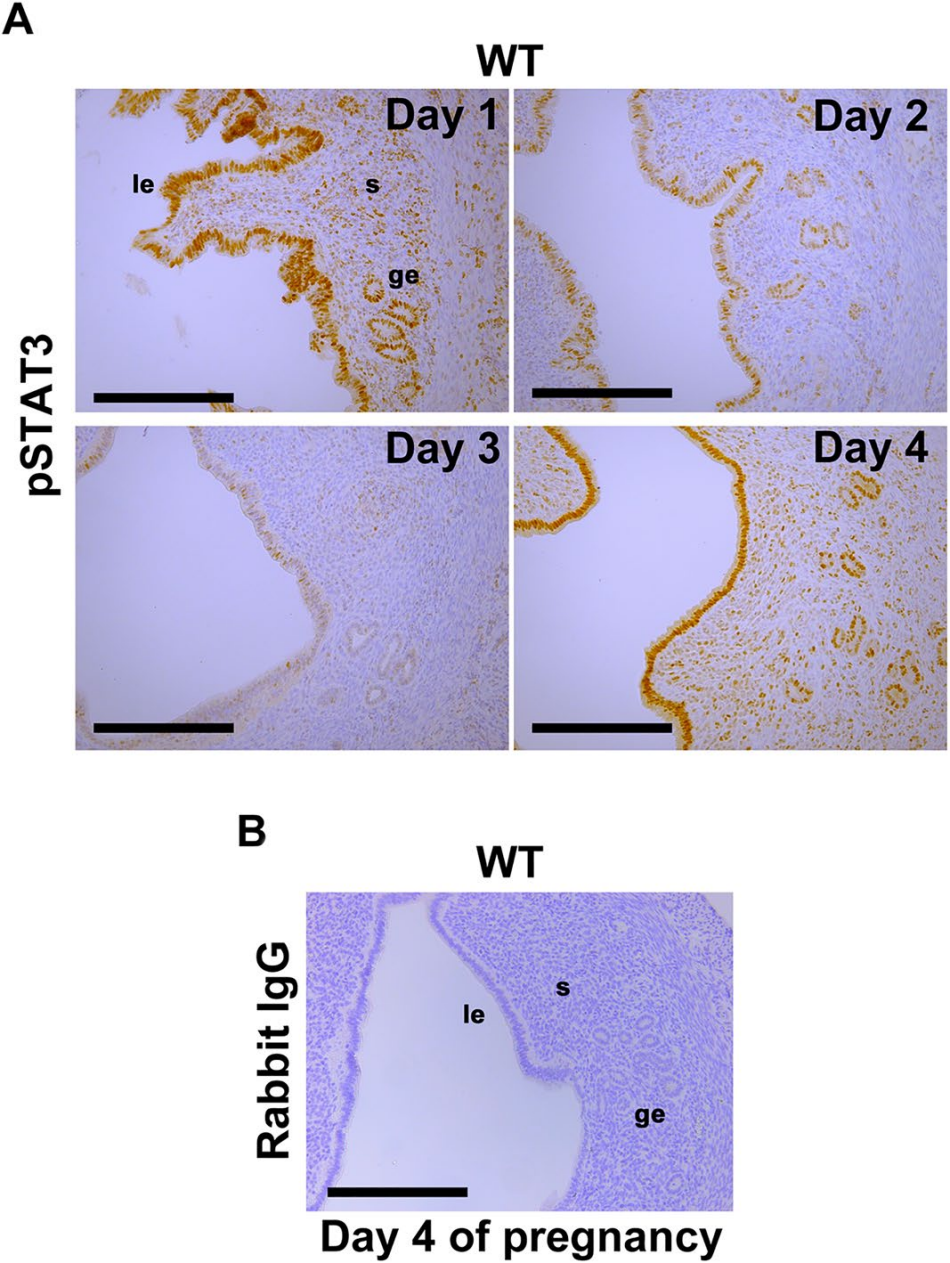
Spatiotemporal activation of STAT3 in the mouse uterus during preimplantation period. (**A**) Phosphorylated STAT3 (pSTAT3), the active form of STAT3, was localized in the epithelium and stroma of WT mouse uteri on days 1 and 4 of pregnancy, in contrast to the weaker immunoreactivity on day 2 and 3. (**B**) As an isotype control of pSTAT3 immunostaining, rabbit IgG was used. The IgG showed negative immunoreactivity in the WT mouse uterus on day 4 of pregnancy. All experiments were performed with more than three biological replicates. Scale bar = 200 μm. le, luminal epithelium; ge, glandular epithelium; s, stroma.

We examined spatiotemporal activation of STAT3 in the uteri of wild-type (WT) mice on days 1, 2, 3 and 4 of pregnancy. The expression of total STAT3 was observed in both uterine epithelium and stroma from days 1 to 4 of pregnancy (Supplemental Fig. S1). The immunostaining for pSTAT3 showed that the activation of STAT3 in both epithelium and stroma was higher on days 1 and 4 compared to days 2 and 3 (Fig. 1A), while the immunoreactivity of isotype control was negative (Fig. 1B). The activation patterns of STAT3 correlate the expression pattern of leukemia inhibitory factor (LIF)^21^, which is expressed in the uterine epitheliumon days 1 and 4 of pregnancy^14,21,22^ and activates uterine STAT3 pathway to induce embryo implantation in mice^5,6,9^.

### Mice with uterine epithelial deletion of STAT3 are infertile due to embryo attachment failure

Although the significance of STAT3 in the whole uterus in mouse embryo implantation has already been demonstrated in the former study using *Stat3*-uKO mice^14^, the specific functions of STAT3 in the epithelium and stroma is still unclear. Recently, various types of genetically-modified mice are available such as *Ltf-iCre* mice and *Amhr2*-Cre mice^23,24^, which have the specific expression of Cre recombinase in the luminal epithelium and the stroma. Crossing these mice with *Stat3*-floxed mice, we generated *Stat3*-deleted mice in the epithelium, stroma and all uterine layers (*Stat3*-eKO, *Stat3*-sKO and *Stat3*-uKO mice, respectively) to clarify the roles of epithelial and stromal STAT3 in embryo implantation.

**Figure 2:**
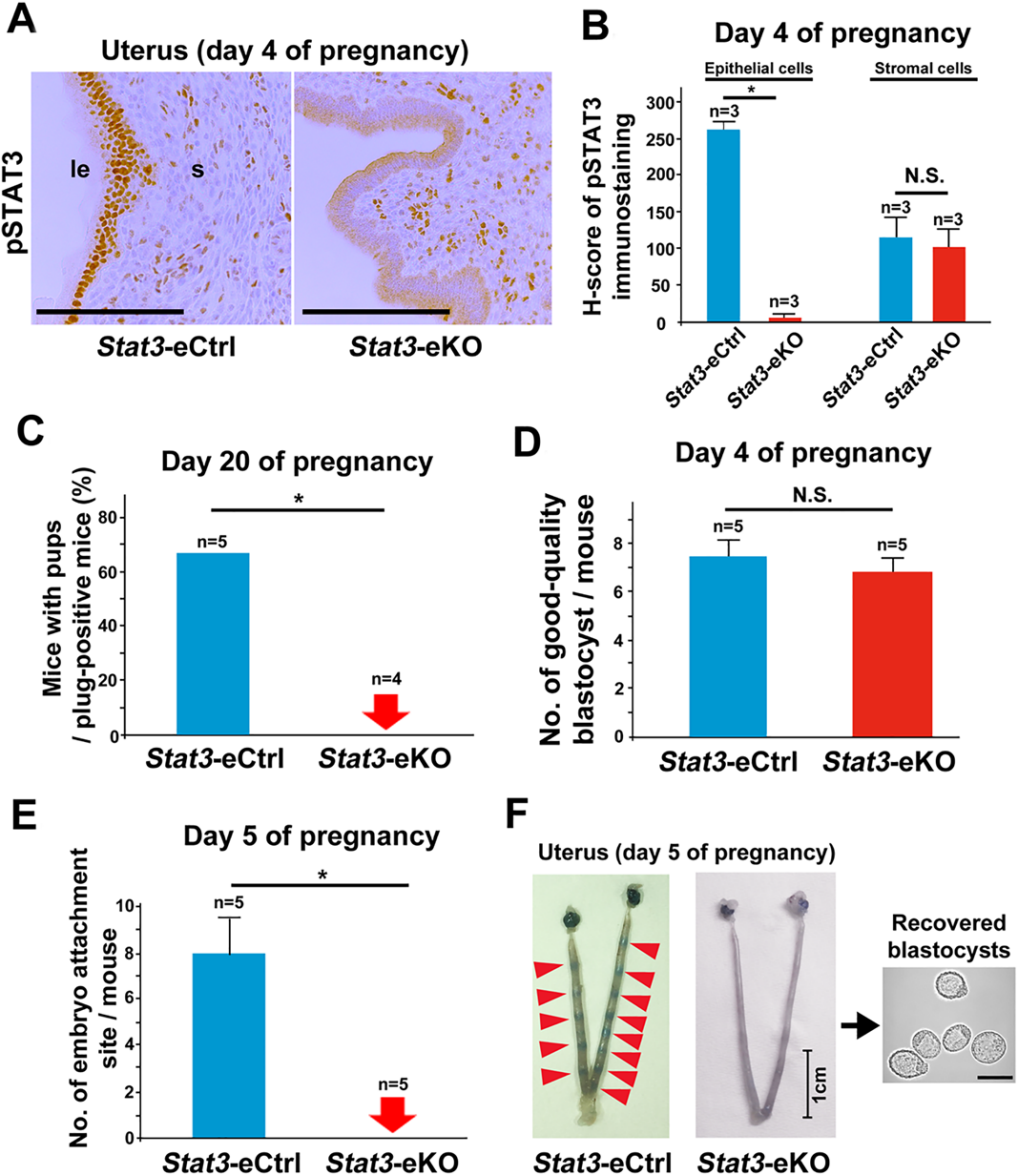
Epithelial deletion of STAT3 leads to embryo attachment failure. (**A, B**) pSTAT3 was efficiently deleted in the uterine epithelium of the mice with epithelial STAT3 deficiency (*Stat3*-eKO mice) on day 4 of pregnancy. *Stat3*-eCtrl means the littermate control mice. Scale bar = 100 μm. le, luminal epithelium; s, stroma. H-scores of pSTAT3 in uterine epithelium and stroma were demonstrated in (**B**). More than three samples obtained from different mice in each group were assessed. (**C**) *Stat3*-eKO females were infertile. Parturition of *Stat3*-eCtrl and *Stat3*-eKO mice were evaluated on day 20 of pregnancy (*, *P* < 0.05, Fisher’s exact probability test). (**D**) The number of good-quality blastocysts retrieved by uterine flushing was comparable between *Stat3*-eCtrl and *Stat3*-eKO mice on day 4 of pregnancy (*P* > 0.05, mean ± SEM, Student’s *t* test). (**E, F**) No embryo attachment sites were observed in *Stat3*-eKO uteri on day 5 of pregnancy (*, *P* < 0.05, mean ± SEM, Mann–Whitney *U* test.). The minimum, median, and maximum values in the numbers of embryo attachment sites in *Stat3*-eCtrl mice were 5, 7, and 13, respectively, and all the mice did not have any attachment sites in *Stat3*-eKO mice. In (**F**), the *Stat3*-eCtrl uterus with 13 attachment sites and the representative *Stat3*-eKO uterus without any attachment sites were presented. The unattached blastocysts were recovered from *Stat3*-eKO uteri by uterine flushing. Scale bar in the uterine pictures = 1 cm. Scale bar in the picture of the recovered embryos = 100 µm. Arrowhead, an embryo attachment site.

We first investigated the reproductive phenotypes of *Stat3*-eKO mice. We performed immunohistochemistry for pSTAT3 and confirmed that the nuclear accumulation of activated STAT3 was clearly diminished in the epithelium of *Stat3*-eKO uteri (Fig. 2A, B). We analyzed the parturition phenotype of *Stat3*-eKO mice to examine the function of epithelial STAT3. *Stat3*-eKO female mice and their littermate controls (*Stat3*-floxed (*Stat3*-eCtrl) mice) were mated with WT fertile male mice. Although vaginal plugs were equally observed both in *Stat3*-eCtrl and *Stat3*-eKO mice, all *Stat3*-eKO mice did not delivered any pups (Fig. 2C). To identify the stage of pregnancy failure in *Stat3*-eKO females, both *Stat3*-eCtrl and *Stat3*-eKO females were sacrificed at the different periods during the early stages of pregnancy. Ovulation and fertilization occur on day 1, and fertilized embryo grows into blastocysts and enter the uterus on day 4 morning. Blastocysts attach to the uterine luminal epithelium from day 4 midnight to day 5 morning^25^. To assess the embryo development before implantation, pregnant *Stat3*-eCtrl and *Stat3*-eKO mice were sacrificed on day 4 morning and bilateral uterine horns were flushed, and the embryos retrieved from uterine lumens were observed with microscope. The numbers of normal-appearing good-quality blastocysts were comparable between *Stat3*-eCtrl and *Stat3*-eKO mice (Fig. 2D), suggesting that ovulation, fertilization and pre-implantation blastocyst growth were normal in *Stat3*-eKO mice. To examine whether these normal blastocysts can attach to the endometrium, *Stat3*-eCtrl and *Stat3*-eKO uteri were examined on day 5. The embryo attachment onto the luminal epithelium induces heightened stromal vascular permeability at the site of the blastocyst and can be visualized by clear blue bands along the uterus after intravenous injection of Chicago blue dye solution^26^. We found that while *Stat3*-eCtrl females had distinct blue bands, *Stat3*-eKO female mice did not have any blue bands (Fig. 2E, F). The unattached blastocysts were recovered from *Stat3*-eKO uteri without any attachment sites by uterine flushing (Fig. 2F). These results indicate that *Stat3*-eKO mice are infertile due to embryo attachment failure.

### Mice with uterine stromal deletion of STAT3 are also infertile due to embryo attachment failure

**Figure 3:**
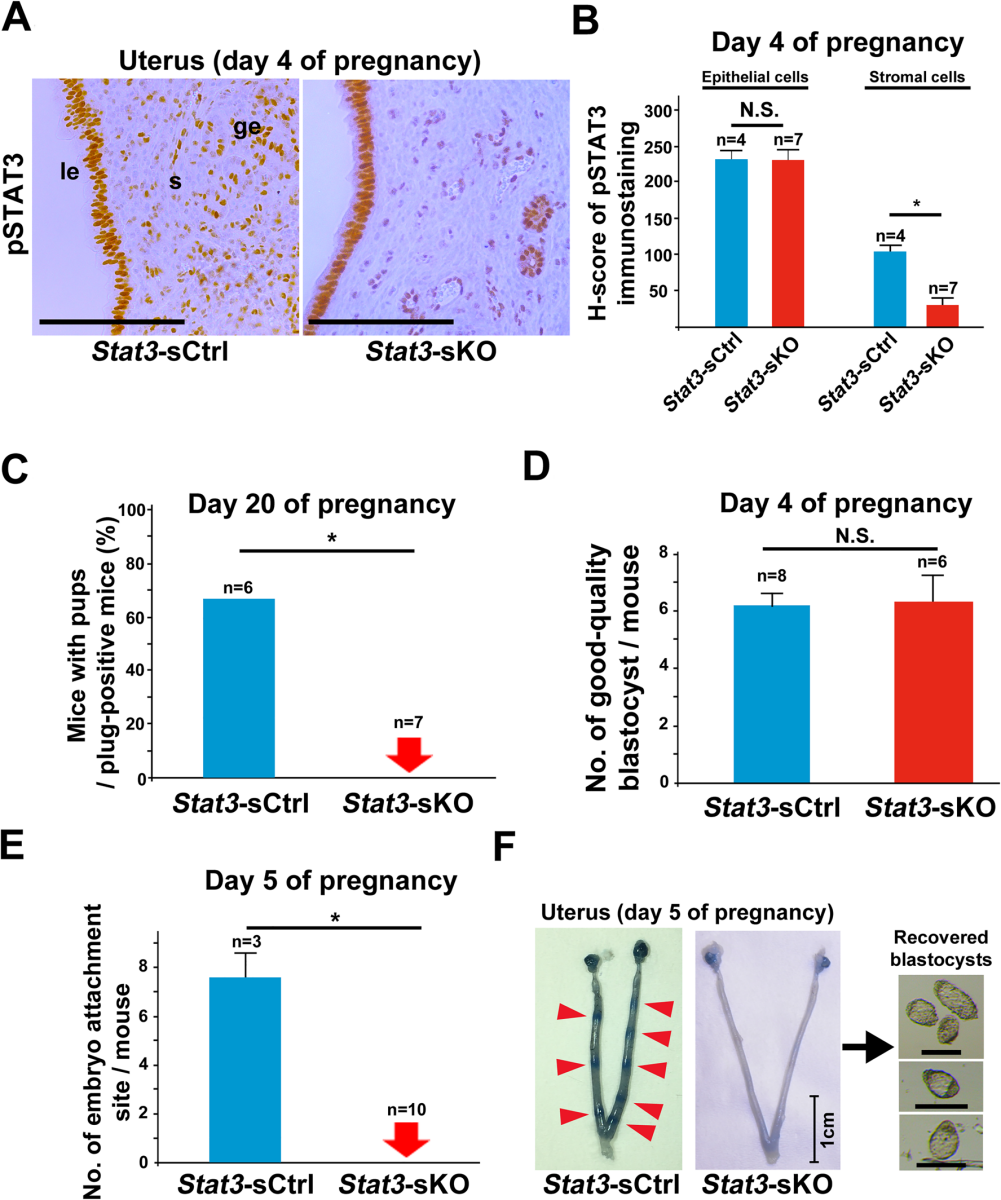
Stromal deletion of STAT3 leads to embryo attachment failure. (**A, B**) pSTAT3 was efficiently deleted in the uterine stroma of the mice with stromal STAT3 deficiency (*Stat3*-sKO mice) on day 4 of pregnancy. *Stat3*-sCtrl means the littermate control mice. Scale bar = 100 μm. le, luminal epithelium; ge, glandular epithelium; s, stroma. H-scores of pSTAT3 in uterine epithelium and stroma were demonstrated in (**B**). More than three samples obtained from different mice in each group were assessed. (**C**) *Stat3*-sKO females were infertile. Parturition of *Stat3*-sCtrl and *Stat3*-sKO mice were evaluated on day 20 of pregnancy (*, *P* < 0.05, Fisher’s exact probability test). (**D**) The number of good-quality blastocysts retrieved by uterine flushing was comparable between *Stat3*-sCtrl and *Stat3*-sKO mice on day 4 of pregnancy (*P* > 0.05, mean ± SEM, Student’s *t* test). (**E, F**) No embryo attachment sites were observed in *Stat3*-sKO uteri on day 5 of pregnancy (*, *P* < 0.05, mean ± SEM, Mann–Whitney *U* test). The minimum, median, and maximum values in the numbers of embryo attachment sites in *Stat3*-sCtrl mice were 6, 8, and 9, respectively, and all the mice did not have any attachment sites in *Stat3*-sKO mice. In Fig. 3F, the *Stat3*-sCtrl uterus with 8 attachment sites and the representative *Stat3*-sKO uterus without any attachment sites were presented. The unattached blastocysts were recovered from *Stat3*-sKO uteri by uterine flushing. Scale bar in the uterine pictures = 1 cm. Scale bar in the picture of the recovered embryos = 100 µm. Arrowhead, an embryo attachment site.

To clarify the role of uterine stromal STAT3 in pregnancy, we next examined *Stat3*-sKO female mice. We performed pSTAT3 immunohistochemistry and confirmed negative staining of pSTAT3 in the stroma of *Stat3*-sKO uteri (Fig. 3A). H-scores of pSTAT3 immunostaining showed significant inactivation of STAT3 in the stroma (Fig. 3B). We analyzed the parturition phenotype of *Stat3*-sKO mice. *Stat3*-sKO female mice and their littermate controls (*Stat3*-floxed (*Stat3*-sCtrl) mice) were mated with WT fertile males. As is the case with *Stat3*-eKO females, all *Stat3*-sKO females failed to produce offspring although vaginal plugs were normally observed (Fig. 3C). Normal-appearing good-quality blastocysts were recovered from *Stat3*-sKO uteri on day 4 of pregnancy (Fig. 3D), but no embryo attachment sites were detected on day 5 with the blue dye method (Fig. 3E, F). The unattached blastocysts were recovered from *Stat3*-sKO uteri without any attachment sites by uterine flushing (Fig. 3F). These findings indicate that *Stat3*-sKO mice are infertile due to embryo attachment failure. Taken together, not only epithelial but also stromal STAT3 is crucial for embryo attachment. However, uterine mRNA expressions of *Lif*, the inducer of embryo attachment through STAT3 activation, were normal in *Stat3*-eKO and *Stat3*-sKO mice (Supplemental Fig. S2), suggesting that the embryo attachment failure in *Stat3*-eKO and *Stat3*-sKO mice is due to the causes other than their uterine LIF expression levels.

### Stromal STAT3 suppresses excessive estrogenic responses in the peri-implantation uterus

**Figure 4:**
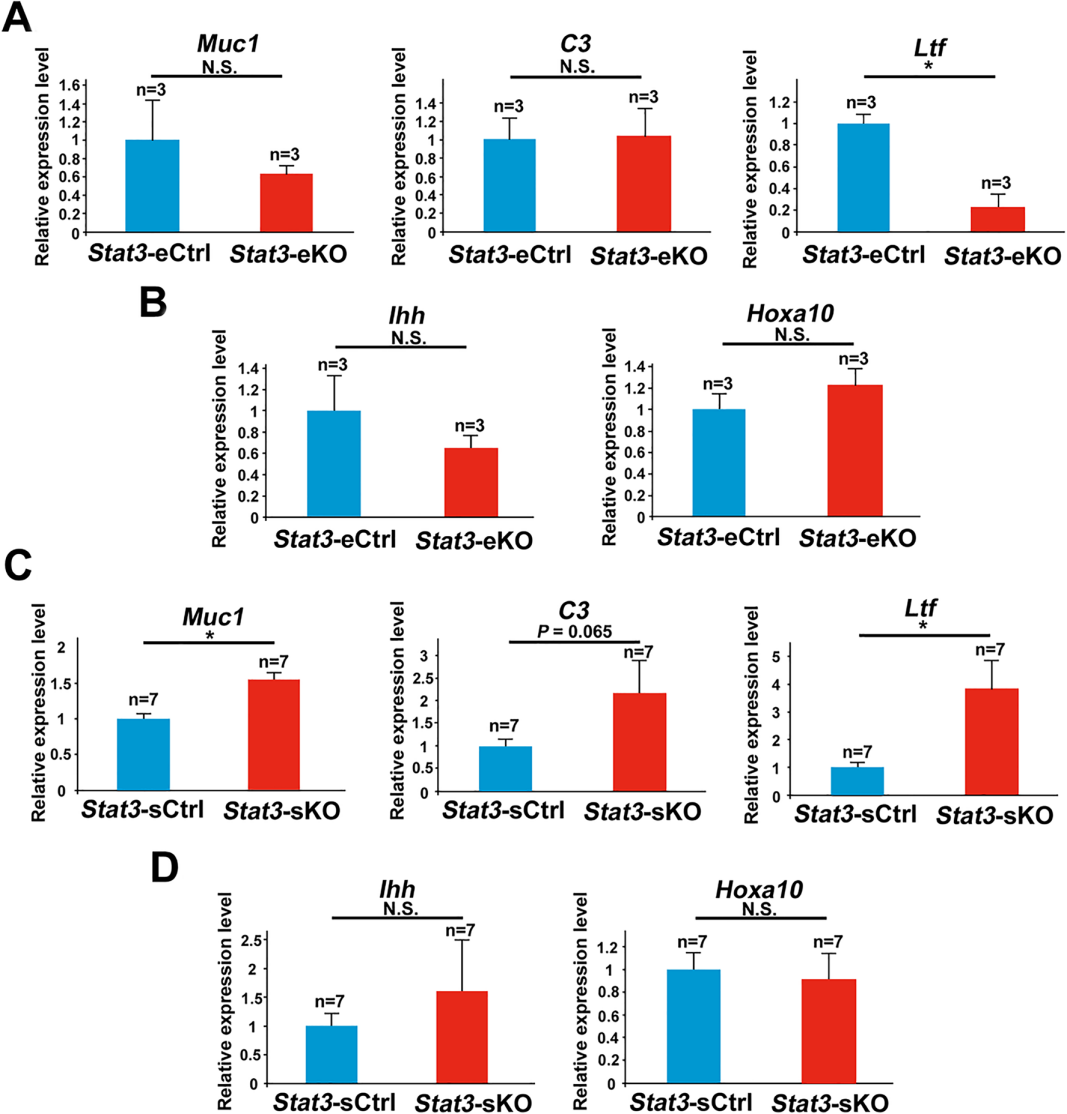
Stromal deletion of STAT3 enhances estrogenic responses in the peri-implantation uterus. (**A, B**) The mRNA expression levels of E_2_ responsive genes *Muc1, C3,* and *Ltf* and P_4_ responsive genes *Ihh* and *Hoxa10* in *Stat3*-eCtrl and *Stat3*-eKO uteri were examined by qPCR. (**C, D**) E_2_ responsive genes *Muc1, C3 and Ltf* and P_4_ responsive genes *Ihh* and *Hoxa10* in *Stat3*-sCtrl and *Stat3*-sKO uteri were evaluated by qPCR (*, *P* < 0.05, mean ± SEM, Student’s *t* test). All assays were performed with more than three biological replicates derived from different mice.

**Figure 5:**
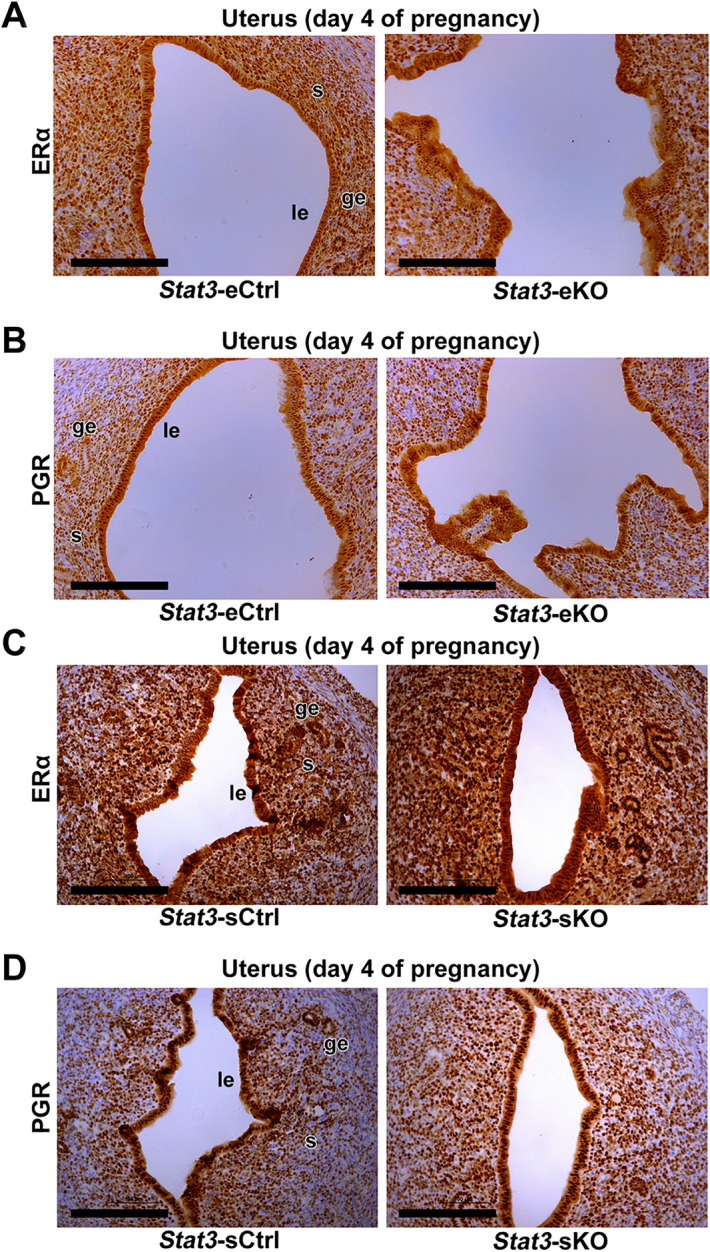
The expressions of uterine ERα and PGR are normal in *Stat3*-eKO and *Stat3*-sKO female mice. **(A**) The immunoreactivities of ERα were comparable in *Stat3*-eCtrl and *Stat3*-eKO uteri. (**B**) The immunoreactivities of PGR were comparable in *Stat3*-eCtrl and *Stat3*-eKO uteri. (**C**) The immunoreactivities of ERα were comparable in *Stat3*-sCtrl and *Stat3*-sKO uteri. (**D**) The immunoreactivities of PGR were comparable in *Stat3*-sCtrl and *Stat3*-sKO uteri. Scale bar = 200 μm. le, luminal epithelium; ge, glandular epithelium; s, stroma.

To elucidate why *Stat3*-eKO and *Stat3*-sKO females exhibit implantation failure, we examined the mRNA expression of genes regulated by 17β-estradiol (E_2_) and progesterone (P_4_). Former investigations have clarified that appropriate balance between E_2_ and P_4_ signaling is a crucial factor for uterine receptivity^1–4,25,27,28^. Therefore, we performed quantitative PCR and examined the expression of *Muc1, C3,* and *Ltf* as E_2_-responsive genes in the epithelium, Indian hedgehog (*Ihh*) and *Hoxa10* as P_4_-responsive genes in the epithelium and stroma on day 4 of pregnancy. As a result, *Stat3*-eKO uteri did not show significant differences in the mRNA levels of neither E_2_-responsive genes nor P_4_-responsive genes compared to *Stat3*-eCtrl ones, except the significant suppression of *Ltf* in *Stat3*-eKO uteri. (Fig. 4A, B). This reduction of *Ltf* mRNA might be affected by *Ltf* promoter-driven Cre recombinase induction in *Stat3*-eKO uteri. On the other hand, *Stat3*-sKO uteri respectively exhibited 1.6, 2.2 and 3.9 times higher mRNA expressions of E_2_-responsive genes *Muc1, C3,* and *Ltf* than *Stat3*-sCtrl ones, while the expression levels of P_4_-responsive genes were unchanged in *Stat3*-sKO uteri compared to *Stat3*-sCtrl ones (Fig. 4C, D). As with *Muc1* mRNA levels, the immunoreactivity of MUC1 was more intense in the luminal epithelium of *Stat3*-sKO mice than in that of *Stat3*-sCtrl mice (Supplemental Fig. S3). In addition, the immunoreactivity of E_2_ receptor ERα and P_4_ receptor PGR were normal in *Stat3*-eKO and *Stat3*-sKO uteri (Fig. 5A–D). These findings suggest that stromal STAT3 suppresses excessive estrogenic responses in the peri-implantation uterus to support uterine receptivity.

### Stromal STAT3 suppresses cell proliferation in the luminal epithelium and epithelial STAT3 forms slit-like structure of uterine lumen in the peri-implantation uterus

**Figure 6:**
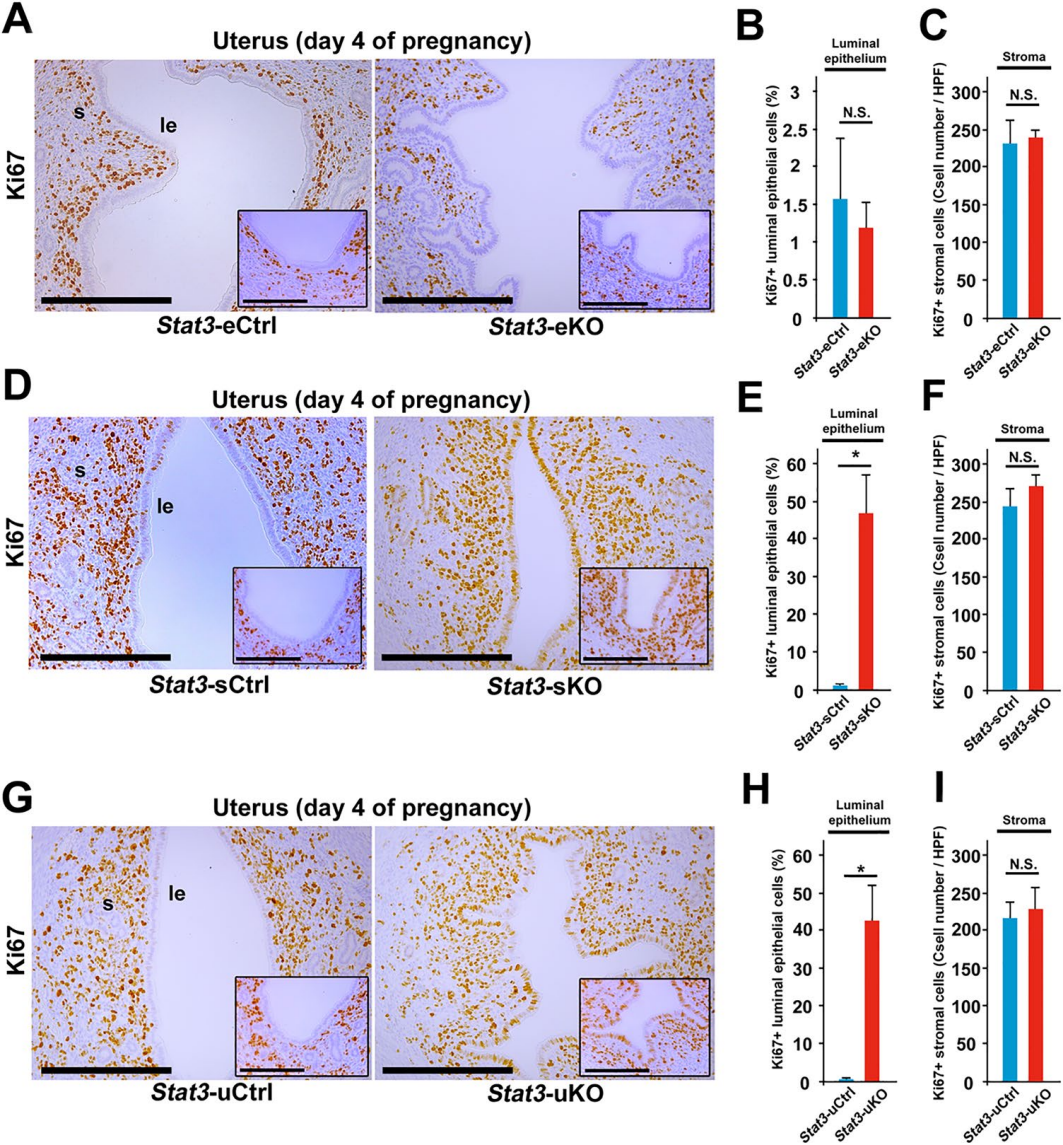
Stromal STAT3 suppresses cell proliferation of luminal epithelium and epithelial STAT3 forms slit-like structure of uterine lumen in the peri-implantation period. **(A**) Cessation of epithelial proliferation occurred normally but slit-like structure of uterine lumen was impaired in *Stat3*-eKO uteri on day 4 of pregnancy. (**B, C**) Percentage of Ki67-positive (Ki67 +) luminal epithelial cells and total number of Ki67 + stromal cells were unchanged between *Stat3*-eCtrl and *Stat3*-eKO uteri (*P* > 0.05, mean ± SEM, Student’s *t* test). (**D**) Slit-like structure of uterine lumen was normal, but persistent epithelial proliferation took place in *Stat3*-sKO uteri on day 4. (**E**) Percentage of Ki67 + luminal epithelial cells were significantly increased in *Stat3*-sKO uteri (*, *P* < 0.05, mean ± SEM, Student’s *t* test). (**F**) Total number of Ki67 + stromal cells were not significantly different between *Stat3*-eCtrl and *Stat3*-eKO uteri (P > 0.05, mean ± SEM, Student’s *t* test). (**G**) Neither slit-like structure of uterine lumen nor cessation of epithelial proliferation was observed in *Stat3*-uKO uteri on day 4. (**H**) Percentage of Ki67 + luminal epithelial cells were significantly increased in *Stat3*-uKO uteri (*, *P* < 0.05, mean ± SEM, Student’s *t* test). (**I**) Total number of Ki67 + stromal cells were not significantly different between *Stat3*-eCtrl and *Stat3*-eKO uteri (*P* > 0.05, mean ± SEM, Student’s *t* test). Scale bar = 200 μm. The antimesometrial edges of uterine lumen were shown in the inset. Scale bar in the inset = 100 µm. le, luminal epithelium; s, stroma.

In the peri-implantation mouse uterus, the luminal epithelial cells undergo cessation of proliferation and the stromal cells have increased proliferative activity, as we call “proliferation-differentiation switching (PDS)” in embryo implantation^1,3,4,6^. PDS is mainly influenced by the balance of E_2_ and P_4_ signaling and is thought to be a critical determinant for uterine receptivity^4,14,27,29^. To investigate the detailed mechanisms of implantation failure in *Stat3*-eKO and *Stat3*-sKO mice, we next assessed PDS in *Stat3*-eKO and *Stat3*-sKO uteri by Ki67 immunostaining on day 4 of pregnancy. In *Stat3*-eKO uteri, most of the luminal epithelial cells were Ki67-negative, and many of the stromal cells were Ki67-posivite, which were comparable with *Stat3*-eCtrl uteri (Fig. 6A–C). However, in *Stat3*-sKO uteri, the numbers of Ki67-positive cells were significantly larger than those in *Stat3*-sCtrl ones (Fig. 6D–F), which might reflect the elevated E_2_ signaling in *Stat3*-sKO uteri (Fig. 4C), while the number of Ki67-positive stromal cells was unchanged between *Stat3*-sCtrl and *Stat3*-sKO uteri (Fig. 6D–F). In addition, aberrant PDS found in *Stat3*-sKO mice was similar to *Stat3*-uKO mice (Fig. 6G–I) which had hyper-estrogenic responses as described in the previous study^14^. Taken together, stromal STAT3 may suppress epithelial estrogenic responses and cease cell proliferation to acquire uterine receptivity and the subsequent embryo attachment.

To clarify the mechanisms of embryo attachment failure despite normal PDS in *Stat3*-eKO mice, we focused on the morphology of uterine lumen. Previous studies have revealed that the slit-like structure of uterine lumen before embryo attachment is critical for normal embryo attachment^30–32^. Histological sections on day 4 showed that the uterine lumen was complicated and indented in both *Stat3*-eKO and *Stat3*-uKO uteri but not in *Stat3*-sKO ones (Fig. 6A, D, G). These findings indicate that uterine epithelial STAT3 contributes to the formation of slit-like luminal structure, which may help successful embryo attachment. To get insight into the mechanism of inappropriate uterine luminal formation in *Stat3*-eKO uteri, we examined the mRNA expression of *Cdh1*, *Wnt5a*, *Msx1* and *Ror2*, the genes associated with appropriate formation of slit-like structure in uterine lumen during peri-implantation period^29,30^. There were no significant differences of these gene expressions between *Stat3*-eCtrl and *Stat3*-eKO uteri (Supplemental Fig. S4), indicating the presence of unknown targets of epithelial STAT3 to control the formation of lumen structure for successful embryo implantation.

## Discussion

**Figure 7:**
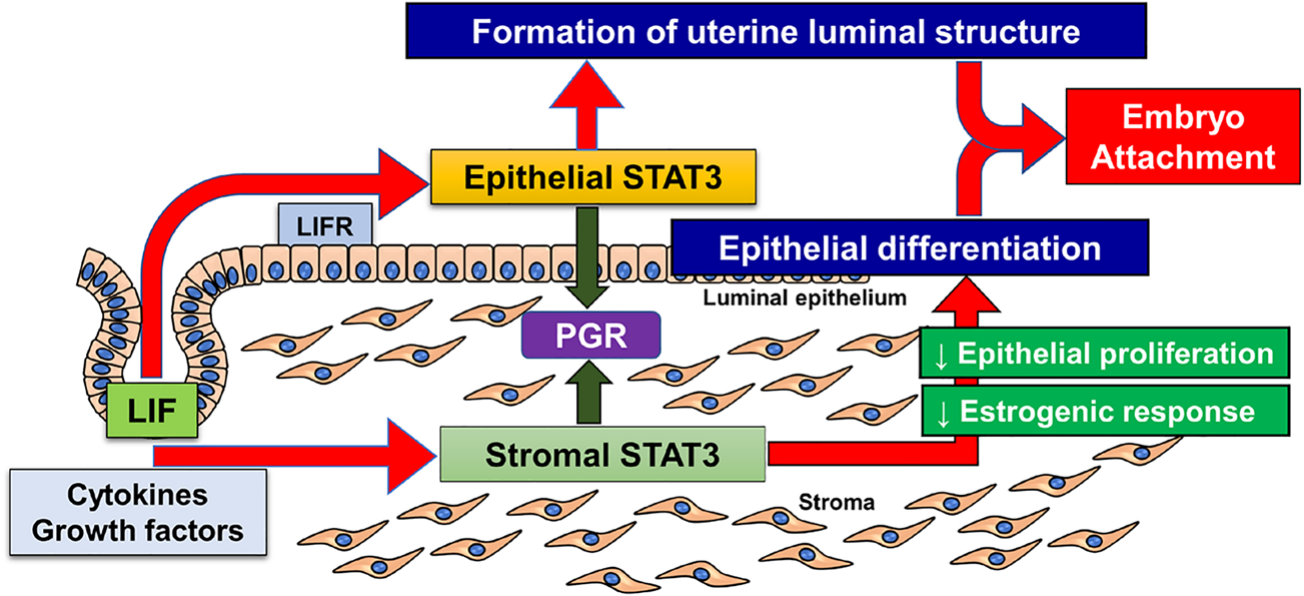
Schematic illustration of the role of uterine epithelial and stromal STAT3 in uterine receptivity and embryo attachment. Epithelial STAT3 regulates the formation of slit-like structure in uterine lumen, and stromal STAT3 suppresses proliferative activity and estrogenic response in the epithelium, thus controlling uterine receptivity and embryo attachment. PGR, progesterone receptor; LIF, leukemia inhibitory factor; LIFR, LIF receptor.

Although a previous study has reported that *Stat3*-uKO female mice show implantation failure due to hyper-estrogenic responses^14^, it has not been elucidated how uterine STAT3 is responsible for uterine receptivity. In the present study, we newly developed two lines of genetically modified mice with epithelial and stromal deletion of STAT3, and found that both epithelial and stromal STAT3 plays essential roles in uterine receptivity and embryo attachment through the different pathways. Epithelial STAT3 controls the formation of slit-like structure of the peri-implantation uterine lumen. On the other hand, stromal STAT3 suppresses E_2_ responsiveness and epithelial proliferation (Fig. 7).

As shown in this study, STAT3 is activated in both epithelium and stroma on days 1 and 4 of pregnancy, LIF is expressed in the luminal and glandular epithelium on day 1 and expressed in the glandular epithelium on day 4, and *Lif* null female mice exhibit infertility due to implantation failure^5^. LIF receptor is localized in the luminal epithelium on day 4 of pregnancy^9,14^. These findings indicate that LIF is a major ligand to trigger epithelial STAT3 activation, although the precise molecular mechanisms how LIF induces embryo implantation remains to be elucidated. In contrast to epithelial STAT3 activation, the previous study showed that the stromal expression of LIF receptor (LIFR) looks faint^14^, implying that LIF-LIFR pathway is not responsible for the stromal STAT3 activation. This speculation may account for the current findings of different functions of epithelial and stromal STAT3.

LIFR is strongly expressed in the epithelium on day 4 of pregnancy^14^, suggesting that dysfunction of uterine LIF-LIFR-STAT3 signaling pathway may affect the phenotypes of *Stat3*-eKO mice such as indented uterine lumen and poor formation of slit-like structure. The previous studies reported that the indented luminal structure is involved in defective implantation^29,30,33^. These findings indicate that LIF-LIFR-STAT3 signaling in the uterine epithelium may control appropriate formation of luminal structure for successful embryo implantation.

A previous study has revealed that entire uterine deletion of *Stat3* using *Stat3*-uKO mice causes the reduction of stromal PGR expression in the peri-implantation uterus^7^. However, stromal PGR expression was not decreased in both *Stat3*-eKO and *Stat3*-sKO mice in our study. These findings make us speculate that both epithelial and stromal STAT3 play a role in stromal PGR expression, and that even if either epithelial or stromal STAT3 is inactivated, stromal PGR is sufficiently maintained. In another previous study of uterine epithelial *Stat3*-deleted mouse model, *Stat3*-floxed *Wnt7a*-Cre (*SW*^*d/d*^) mice show the increased expression of E-cadherin encoded by *Cdh1*^34^. Our study demonstrated that both stromal proliferation and uterine *Cdh1* expression are normal in *Stat3*-floxed *Ltf*-iCre (*Stat3*-eKO) mice, another mouse model of uterine epithelial deletion of *Stat3*. There are temporal differences in epithelial Cre recombinase induction between *Ltf*-iCre and *Wnt7a*-Cre mice. *Wnt7a* is expressed in the uterine epithelium from fetal and neonatal stages^35^, while *Ltf* is expressed from the postpubertal stage^23^. These findings indicate that epithelial STAT3 in the developmental stage may suppress E-cadherin expression.

Also, a previous study has reported that *Stat3*-floxed/*Amhr2-Cre* female mice were subfertile due to resorption, although embryo implantation was normal^36^. In the present study, *Stat3*-floxed/*Amhr2-Cre* mice showed implantation failure. The discrepancy of the phenotypes between the previous and present studies might be derived from different *Stat3-*floxed mouse lines in which loxP-sites are inserted into the different loci. Therefore, it is possible that different efficiency of *Stat3* deletion might cause the inconsistent phenotypes between these studies.

The present study showed that *Stat3*-sKO uteri have the enhanced estrogenic responses in the epithelium, suggesting the stromal-epithelial interactions through stromal STAT3. There is accumulated evidence showing that stromal transcriptional factors such as *Hand2* and *Hif2a* affect epithelial functions to support embryo implantation^37,38^. In this sense, STAT3 could be another stromal transcriptional factor regulating epithelial conditions during embryo implantation.

The interactions between the uterus and embryos provide blastocyst growth and competency to embryo implantation^39,40^. Our study showed that neither epithelial nor stromal *Stat3* deficiency affects blastocyst growth until day 4 of pregnancy. Since epithelial and stromal STAT3 may affect the intrauterine blastocyst growth and the blastocyst competency to embryo attachment from day 4 midnight to day 5, further investigations are needed to elucidate this issue.

The present study revealed that both epithelial and stromal STAT3 are critical for uterine receptivity and embryo attachment in different manners. However, it remains elusive how epithelial STAT3 regulates the formation of slit-like structure in uterine lumen, and how stromal STAT3 controls estrogen responsiveness in the epithelium. Although uterine STAT3 is involved in the uterine regeneration process^15^, it is also unclear whether regeneration activity of uterine STAT3 is associated with embryo implantation. Further investigations are needed to answer these questions and to clarify the association of STAT3 with recurrent implantation failure in humans.

## Methods

### Mice

WT (C57BL/6 N), *Stat3*-floxed mice (Oriental Bio Service, Kyoto, Japan)^17^, *Ltf-iCre* mice^23^, *Amhr2-Cre* mice^24^, and *Pgr-Cre* mice^16^ were used in this study. *Ltf*, *Amhr2* and *Pgr* are expressed in the luminal epithelium, the stroma, and all layers of the endometrium, respectively. Therefore, *Ltf-iCre*, *Amhr2-Cre* and *Pgr-Cre* mice have the specific expression of Cre recombinase in the luminal epithelium, the stroma, and the endometrium, respectively. We generated *Stat3*-floxed/*Ltf-iCre* (*Stat3*-eKO), *Stat3*-floxed/*Amhr2-Cre* (*Stat3*-sKO), and *Stat3*-floxed/*Pgr-Cre* (*Stat3*-uKO) mice to deplete *Stat3* specifically in the luminal epithelium, the stroma, and the endometrium, respectively. For the experiments of pregnancy, female mice were mated with fertile WT males. Day 1 of pregnancy was defined as the day when we recognized vaginal plug. These female mice were sacrificed on days 1, 2, 3, 4 and 5 for the evaluation of embryo implantation and sample collection. Embryo attachment sites were identified as blue bands under the intravenous injection of Chicago blue dye solution^26^. When no embryo attachment sites were detected, uterine horns were cut and flushed with normal saline to obtain embryos to confirm whether embryo development and transportation from the oviduct into the uterus were normal. All mice were housed in the University of Tokyo Animal Care Facility according to the institutional guidelines for the use of laboratory animals. All animal experiments were approved by the Institutional Animal Experiment Committee of the University of Tokyo Graduate School of Medicine (Approval number P16-066).

### Immunohistochemistry

Immunostaining was performed in 10% formalin-fixed paraffin-embedded sections (6 µm), using antibodies to Ki67 (Thermo Fisher Scientific, SP6), MUC1 (Abcam, ab15481), ERα (Abcam, ab810922), PGR (Abcam, ab63605), total STAT3 (Santa Cruz, sc-8019) and phosphorylated STAT3 (phospho Y705, Abcam, ab76315). As an isotype control, rabbit IgG (Dako, IS600) was used.

### H-score

The immunoreactivity of pSTAT3 and MUC1 staining was evaluated by a semiquantitative H-score^41^. The intensity of each cell was graded with a value of 0, 1+, 2+, or 3+ (negative, weak, moderate, or strong, respectively) in a high-power field, and then, the percentage of cells at each staining intensity level was calculated. H-score was obtained by the following equation: H-score = 1 × (% cells 1 +)+ 2 × (% cells 2 +) + 3 × (% cells 3 +) Five high-powered fields per respective section were analyzed, and the average value was assigned as the H-score for each section from different mice.

### Evaluation of PDS

Ki67-positive (Ki67 +) epithelial and stromal cell number on five selected high-power fields in the uterine sections obtained from more than three different mice in each group were manually counted. PDS was assessed by the percentage of Ki67 + luminal epithelial cells and the total number of Ki67 + stromal cells.

### RNA extraction quantitative PCR (qPCR)

Total RNA extraction was performed as described previously^4,6,38,42,43^. The complementary DNA were synthesized from the extracted RNA using ReverTra Ace qPCR RT Master Mix (TOYOBO), and, and qPCR was performed using SYBR Green PCR Master Mix (Thermo Fisher Scientific). A housekeeping gene *Actb* was used as an internal standard for normalizing the relative mRNA expression. Sequences of qPCR primers which were used to detect each gene are shown in Table 1.

**Table 1:** Sequences of qPCR primers.

Gene	Forward	Reverse
*Actb*	TGTTACCAACTGGGACGACA	GGGGTGTTGAAGGTCTCAAA
*C3*	TGTTACCAACTGGGACGACA	GGGGTGTTGAAGGTCTCAAA
*Cdh1*	AGGTTTTCGGGCACCACTTA	TGATGTTGCTGTCCCCAAGT
*Hoxa10*	TAACTTAGCCGGAGCCTTAGGTC	CCTGATTAAACACAGCCCAGCA
*Ihh*	GAGAACACGGGTGCCGACCG	CAGCGGCCGAATGCTCAGACT
*Lif*	GCCCTGTAAATGCCACCTGT	CGACCATCCGATACAGCTCC
*Ltf*	GGAGCCTTGAGGTGTCTGAG	CCAGGTGGCACTCCTTGTAT
*Msx1*	CCAGCCAGACGGCTGAGTC	GGACCGCCAAGAGGAAAAGA
*Muc1*	GTGCCAGTGCCGCCGAAAGA	CCGCCAAAGCTGCCCCAAGT
*Ror2*	GACCGGTTTGGCAAGGTCTA	GACCAGGAACTCATGGAGGT
*Wnt5a*	CATCGACTATGGCTACCGCTTC	CACTCCATGACACTTACAGGCTACA

### Statistical analysis

Statistical analyses were performed using two-tailed Student’s *t* test, Mann–Whitney U test, and Fisher’s exact probability test as appropriate. *P* values less than 0.05 were considered statistically significant.

## Supplementary information

Supplementary Information.

Supplementary Figure S1.

Supplementary Figure S2.

Supplementary Figure S3.

Supplementary Figure S4.

## References

[1] Fukui Y (2019). Uterine receptivity, embryo attachment, and embryo invasion: multistep processes in embryo implantation. Reprod. Med. Biol..

[2] Dey SK (2004). Molecular cues to implantation. Endocr. Rev..

[3] Hirota Y (2019). Progesterone governs endometrial proliferation–differentiation switching and blastocyst implantation. Endocr. J..

[4] Haraguchi H (2014). MicroRNA-200a locally attenuates progesterone signaling in the cervix, preventing embryo implantation. Mol. Endocrinol..

[5] Stewart CL (1992). Blastocyst implantation depends on maternal expression of leukaemia inhibitory factor. Nature.

[6] Matsuo M (2019). Levonorgestrel inhibits embryo attachment by eliminating uterine induction of leukemia inhibitory factor. Endocrinology.

[7] Lee JH (2013). Signal transducer and activator of transcription-3 (Stat3) plays a critical role in implantation via progesterone receptor in uterus. FASEB J..

[8] Hillmer EJ, Zhang H, Li HS, Watowich SS (2016). STAT3 signaling in immunity. Cytokine Growth Factor Rev..

[9] Cheng JG, Chen JR, Hernandez L, Alvord WG, Stewart CL (2001). Dual control of LIF expression and LIF receptor function regulate Stat3 activation at the onset of uterine receptivity and embryo implantation. Proc. Natl. Acad. Sci. U S A.

[10] Taniguchi K (2015). A gp130-Src-YAP module links inflammation to epithelial regeneration. Nature.

[11] Kaur S, Bansal Y, Kumar R, Bansal G (2020). A panoramic review of IL-6: structure, pathophysiological roles and inhibitors. Bioorg. Med. Chem..

[12] Schmetterer KG, Pickl WF (2017). The IL-10/STAT3 axis: contributions to immune tolerance by thymus and peripherally derived regulatory T-cells. Eur. J. Immunol..

[13] Takeda K (1997). Targeted disruption of the mouse Stat3 gene leads to early embryonic lethality. Proc. Natl. Acad. Sci. U S A.

[14] Sun X, Bartos A, Whitsett JA, Dey SK (2013). Uterine deletion of Gp130 or Stat3 shows implantation failure with increased estrogenic responses. Mol. Endocrinol..

[15] Hiraoka, T. et al. STAT3 accelerates uterine epithelial regeneration in a mouse model of decellularized uterine matrix transplantation. *JCI Insight* **1** (2016). 10.1172/jci.insight.87591 PMC4922514 27358915

[16] Soyal SM (2005). Cre-mediated recombination in cell lineages that express the progesterone receptor. Genesis.

[17] Takeda K (1998). Stat3 activation is responsible for IL-6-dependent T cell proliferation through preventing apoptosis: generation and characterization of T cell-specific Stat3-deficient mice. J. Immunol..

[18] Kelleher AM (2017). Forkhead box a2 (FOXA2) is essential for uterine function and fertility. Proc. Natl. Acad. Sci. U S A.

[19] Wang X (2018). SOX17 regulates uterine epithelial-stromal cross-talk acting via a distal enhancer upstream of Ihh. Nat. Commun..

[20] Yuan J (2019). Primary decidual zone formation requires Scribble for pregnancy success in mice. Nat. Commun..

[21] Song H, Lim H, Das SK, Paria BC, Dey SK (2000). Dysregulation of EGF family of growth factors and COX-2 in the uterus during the preattachment and attachment reactions of the blastocyst with the luminal epithelium correlates with implantation failure in LIF-deficient mice. Mol. Endocrinol..

[22] Bhatt H, Brunet LJ, Stewart CL (1991). Uterine expression of leukemia inhibitory factor coincides with the onset of blastocyst implantation. Proc. Natl. Acad. Sci. U S A.

[23] Daikoku T (2014). Lactoferrin-iCre: a new mouse line to study uterine epithelial gene function. Endocrinology.

[24] Jamin SP, Arango NA, Mishina Y, Hanks MC, Behringer RR (2002). Requirement of Bmpr1a for Mullerian duct regression during male sexual development. Nat. Genet..

[25] Wang H, Dey SK (2006). Roadmap to embryo implantation: clues from mouse models. Nat. Rev. Genet..

[26] Das SK (1994). Heparin-binding EGF-like growth factor gene is induced in the mouse uterus temporally by the blastocyst solely at the site of its apposition: a possible ligand for interaction with blastocyst EGF-receptor in implantation. Development.

[27] Tranguch S (2007). FKBP52 deficiency-conferred uterine progesterone resistance is genetic background and pregnancy stage specific. J. Clin. Invest..

[28] Hirota Y (2010). Uterine FK506-binding protein 52 (FKBP52)-peroxiredoxin-6 (PRDX6) signaling protects pregnancy from overt oxidative stress. Proc. Natl. Acad. Sci. U S A.

[29] Daikoku T (2011). Conditional deletion of Msx homeobox genes in the uterus inhibits blastocyst implantation by altering uterine receptivity. Dev. Cell.

[30] Cha J (2014). Appropriate crypt formation in the uterus for embryo homing and implantation requires Wnt5a-ROR signaling. Cell Rep..

[31] Arora R (2016). Insights from imaging the implanting embryo and the uterine environment in three dimensions. Development.

[32] Yuan J (2018). Tridimensional visualization reveals direct communication between the embryo and glands critical for implantation. Nat. Commun..

[33] Yuan J (2016). Planar cell polarity signaling in the uterus directs appropriate positioning of the crypt for embryo implantation. Proc. Natl. Acad. Sci. U S A.

[34] Pawar S (2013). STAT3 regulates uterine epithelial remodeling and epithelial–stromal crosstalk during implantation. Mol. Endocrinol..

[35] Parr BA, McMahon AP (1998). Sexually dimorphic development of the mammalian reproductive tract requires Wnt-7a. Nature.

[36] Robker RL (2014). Identification of sites of STAT3 action in the female reproductive tract through conditional gene deletion. PLoS ONE.

[37] Li Q (2011). The antiproliferative action of progesterone in uterine epithelium is mediated by Hand2. Science.

[38] Matsumoto L (2018). HIF2alpha in the uterine stroma permits embryo invasion and luminal epithelium detachment. J. Clin. Invest..

[39] Paria BC, Huet-Hudson YM, Dey SK (1993). Blastocyst's state of activity determines the "window" of implantation in the receptive mouse uterus. Proc. Natl. Acad. Sci. U S A.

[40] Hamatani T (2004). Global gene expression analysis identifies molecular pathways distinguishing blastocyst dormancy and activation. Proc. Natl. Acad. Sci. U S A.

[41] Hirota Y (2008). Deficiency of immunophilin FKBP52 promotes endometriosis. Am. J. Pathol..

[42] Egashira M (2017). F4/80+ macrophages contribute to clearance of senescent cells in the mouse postpartum uterus. Endocrinology.

[43] Haraguchi H (2019). Mdm2-p53-SF1 pathway in ovarian granulosa cells directs ovulation and fertilization by conditioning oocyte quality. FASEB J..

