# Supplementary material for: Differential roles of uterine epithelial and stromal STAT3 coordinate uterine receptivity and embryo attachment
Source: Sci Rep. 2020 Sep 23;10:15523. doi: 10.1038/s41598-020-72640-0 (PMC7511330; doi:10.1038/s41598-020-72640-0)
Supplement: Supplementary file 1 — Supplementary Information. [file 41598_2020_72640_MOESM1_ESM.docx]

# **Title:** Differential roles of uterine epithelial and stromal STAT3 coordinate uterine receptivity and embryo attachment

**Running title:** Epithelial/stromal STAT3 in implantation

**Authors:** Takehiro Hiraoka^1,2^, Yasushi Hirota^1,3^, Yamato Fukui^1^, Mona Gebril^1^, Tetsuaki Kaku^1^, Shizu Aikawa^1^, Tomoyuki Hirata^1^, Shun Akaeda^1^, Mitsunori Matsuo^1^, Hirofumi Haraguchi^1^, Mayuko Saito-Kanatani^1^, Ryoko Shimizu-Hirota^4^, Norihiko Takeda^5^, Osamu Yoshino^2^, Tomoyuki Fujii^1^, Yutaka Osuga^1^

**Affiliations:**

1. Department of Obstetrics and Gynecology, Graduate School of Medicine, The University of Tokyo, Bunkyo-ku, Tokyo, Japan.

2. Department of Obstetrics and Gynecology, Kitasato University, Sagamihara, Kanagawa, Japan.

3. Frontier Outstanding Research for Clinical Empowerment (FORCE), Japan Agency for Medical Research and Development (AMED), Bunkyo-ku, Tokyo 113-8655, Japan.

4. Department of Internal Medicine, Center of Preventive Medicine, School of Medicine, Keio University, Shinjuku-ku, Tokyo, Japan.

5. Center for Molecular Medicine, Jichi Medical University, Shimotuke, Tochigi, Japan.

**Address correspondence to:** Yasushi Hirota, MD, PhD. Department of Obstetrics and Gynecology, Graduate School of Medicine, The University of Tokyo, 7-3-1 Hongo, Bunkyo-ku, Tokyo 113-8655, Japan.

Phone: 81.3.3815.5411 Fax: 81.3.3816.2017 E-mail: [yhirota-tky@umin.ac.jp](mailto:yhirota-tky@umin.ac.jp)

**Figure Legends of Supplemental Figures**

**Supplemental Fig. S1. Total STAT3 is expressed in the epithelium and stroma of wild-type (WT) mouse uterus during the preimplantation period.** Total STAT3 was constantly expressed in the wild-type (WT) mouse uterine epithelium and stroma on days 1, 2, 3 and 4 of pregnancy. All assays were performed with more than three biological replicates obtained from different mice. Scale bar = 200μm. le, luminal epithelium; ge, glandular epithelium; s, stroma.

**Supplemental Fig. S2. Uterine expression of *Lif mRNA is normal in Stat3*-eKO and *Stat3*-sKO mice on day 4 of pregnancy.** The expression level of *Lif* mRNA in day 4 uteri were comparable between *Stat3*-eCtrl and *Stat3*-eKO mice and between *Stat3*-sCtrl and *Stat3*-sKO mice(*P>*0.05, mean ± SEM, Student’s *t* test). All assays were performed with more than three biological replicates deriving from different mice.

**Supplemental Fig. S3. MUC1 protein is expressed more intensely in the luminal epithelium of *Stat3*-sKO mice on day 4 of pregnancy. A&B**, MUC1 protein was expressed more intensely in the luminal epithelium of *Stat3*-sKO mice than in that of *Stat3*-sCtrl mice on day 4 of pregnancy (*P<*0.05, mean ± SEM, Student’s *t* test). Scale bar = 200μm. le, luminal epithelium; s, stroma. H-scores of MUC1 in the luminal epithelium were demonstrated in **B**. Five samples obtained from different mice in each group were assessed.

**Supplemental Fig. S4. Uterine expression levels of the genes associated with the formation of luminal structure are normal in *Stat3*-eKO mice on day 4 of pregnancy.** The mRNA expression levels of *Cdh1*, *Msx1*, *Ror2* and *Wnt5a*, the genes associated with the formation of slit-like uterine luminal structure, showed no significant differences between *Stat3*-eCtrl and *Stat3*-eKO (*P>*0.05, mean ± SEM, Student’s *t* test). All assays were performed with more than three biological replicates deriving from different mice.
