# Supplementary figures and images for: Differential roles of uterine epithelial and stromal STAT3 coordinate uterine receptivity and embryo attachment
Source: Sci Rep. 2020 Sep 23;10:15523. doi: 10.1038/s41598-020-72640-0 (PMC7511330; doi:10.1038/s41598-020-72640-0)

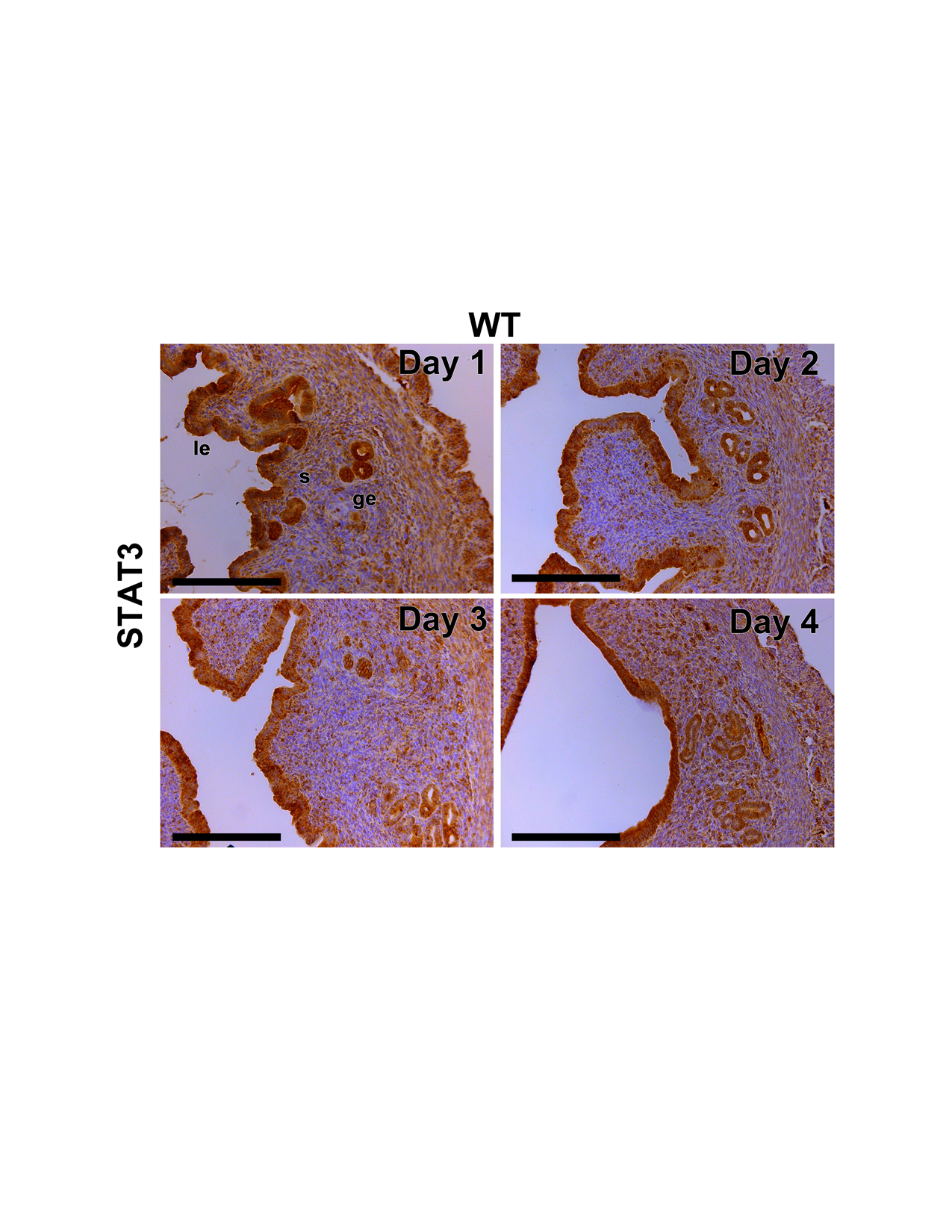

Supplement: Supplementary file 2 — Supplementary Figure S1. [file 41598_2020_72640_MOESM2_ESM.tif]

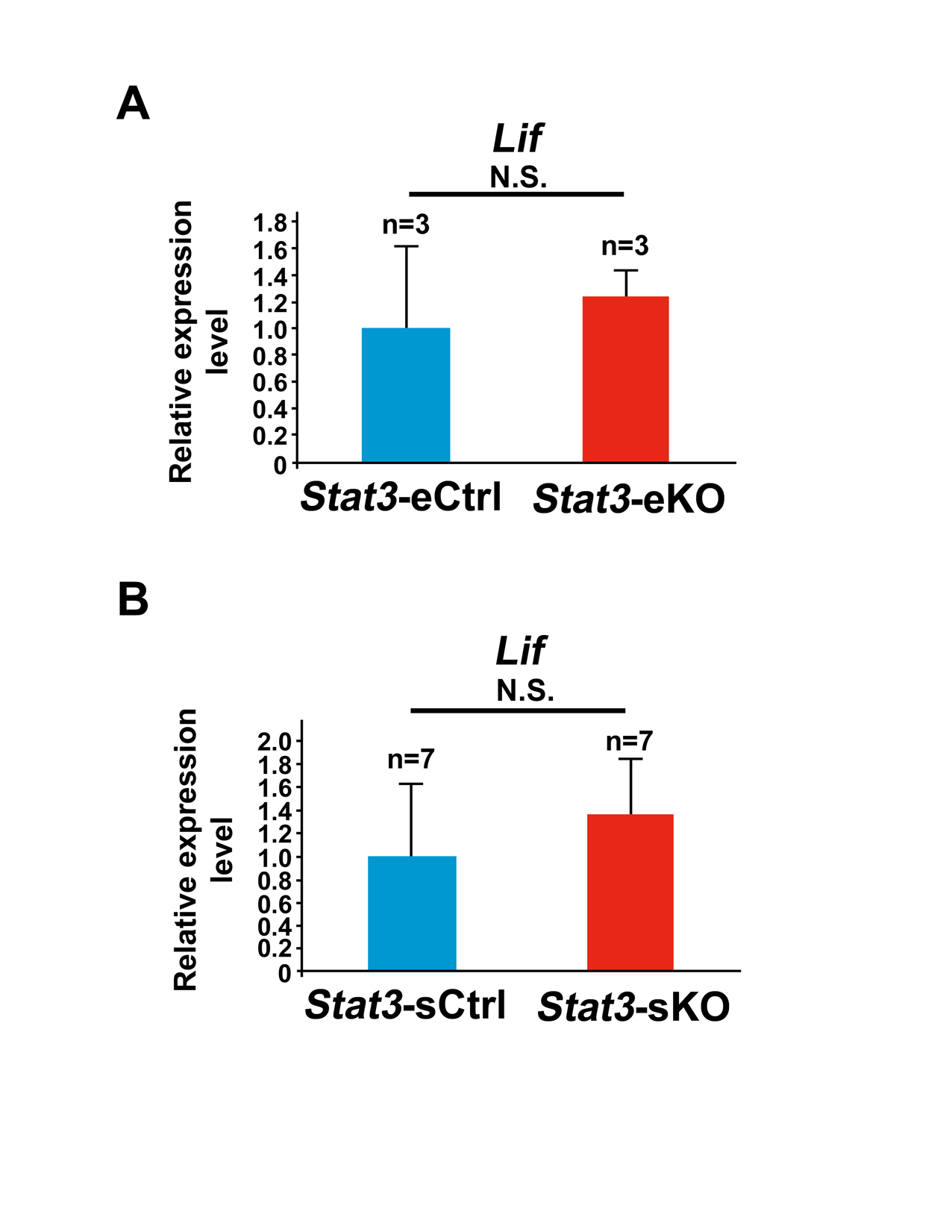

Supplement: Supplementary file 3 — Supplementary Figure S2. [file 41598_2020_72640_MOESM3_ESM.tif]

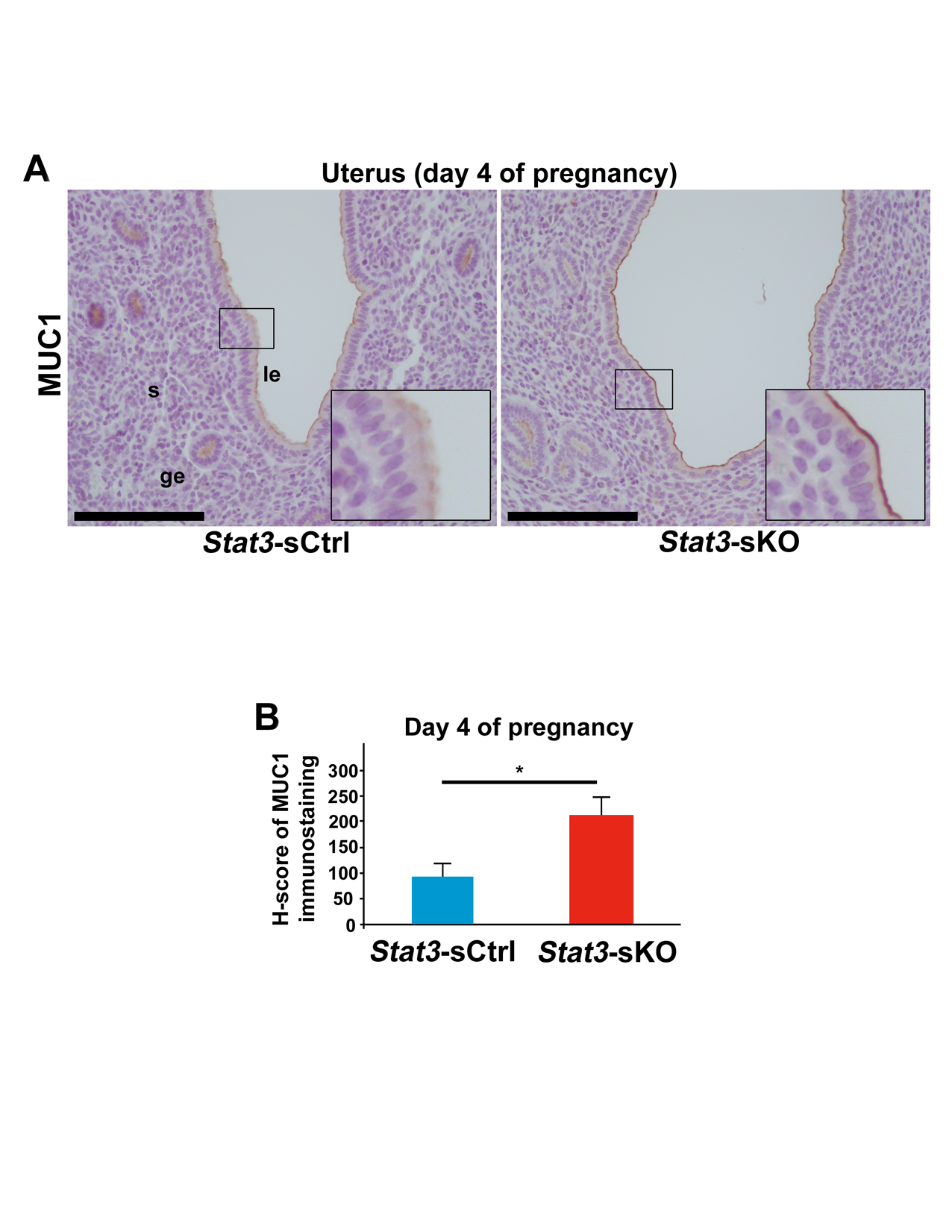

Supplement: Supplementary file 4 — Supplementary Figure S3. [file 41598_2020_72640_MOESM4_ESM.tif]

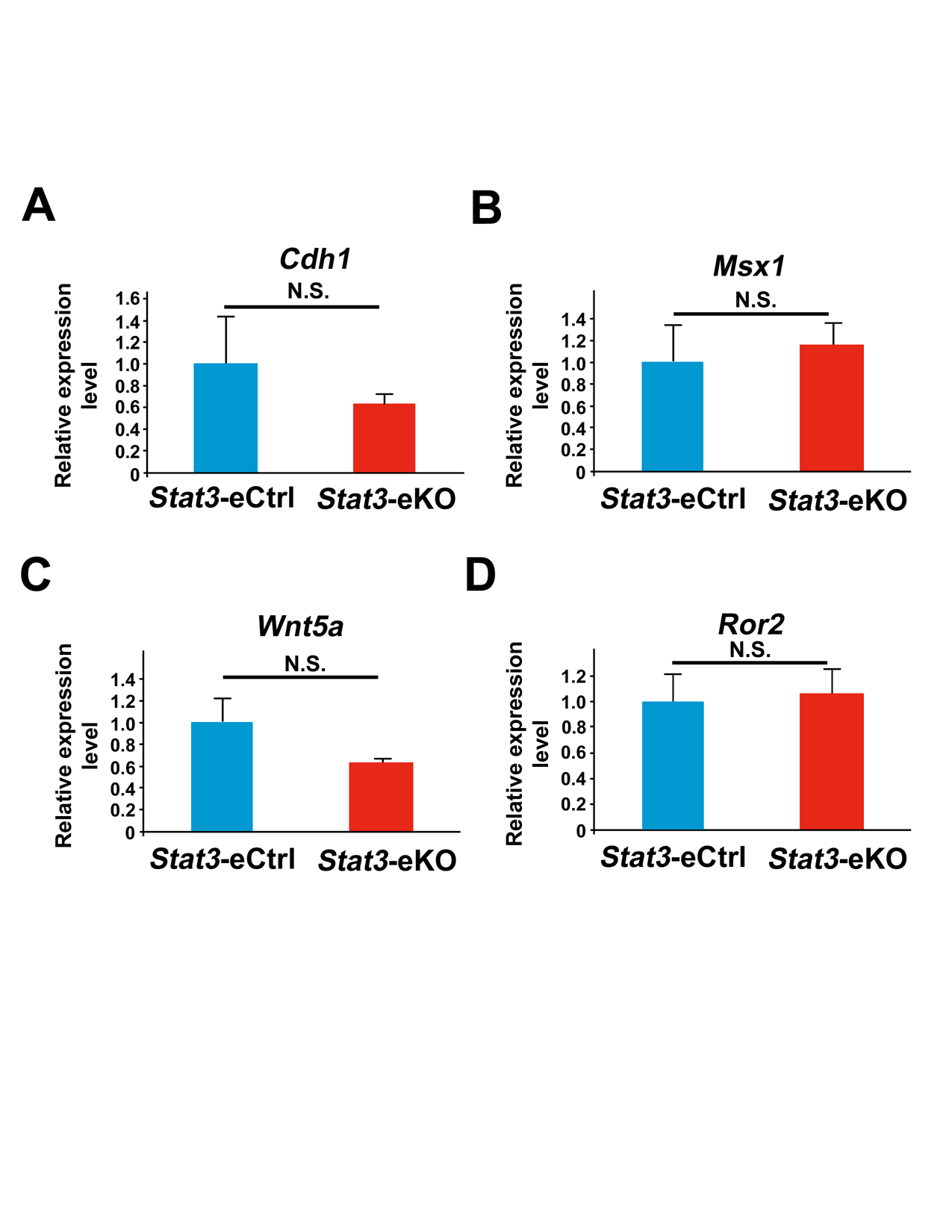

Supplement: Supplementary file 5 — Supplementary Figure S4. [file 41598_2020_72640_MOESM5_ESM.tif]
